# Venous, Arterial, and Neuropathic Leg Ulcers With Emphasis on the Geriatric Population

**DOI:** 10.7759/cureus.38123

**Published:** 2023-04-25

**Authors:** Harvey N Mayrovitz, Summer Wong, Camilla Mancuso

**Affiliations:** 1 Medical Education, Nova Southeastern University Dr. Kiran C. Patel College of Allopathic Medicine, Davie, USA; 2 Dermatology, Nova Southeastern University Dr. Kiran C. Patel College of Osteopathic Medicine, Davie, USA

**Keywords:** chronic venous ulcers, chronic leg ulcers, management of leg ulcers, evaluation of ulcers, neuropathic ulcers, diabetic ulcers, arterial-ischemic ulcers, arterial ulcers, venous ulcers, leg ulcers

## Abstract

Leg ulcers are a common and often serious problem in older adults. Underlying conditions that increase risk include age-related increases in chronic venous insufficiency, peripheral artery disease, connective tissue and autoimmune conditions, reduced mobility, and diabetes mellitus (DM). Geriatric patients have a higher risk of multiple wound-related complications including infection, cellulitis, ischemia, and gangrene, any of which may lead to further complications including amputation. The very presence of these lower extremity ulcers in the elderly negatively impacts their quality of life and ability to function. Understanding and early identification of the underlying conditions and wound features are important for effective ulcer healing and complication mitigation. This targeted review focuses on the three most common types of lower extremity ulcers: venous, arterial, and neuropathic. The goal of this paper is to characterize and discuss the general and specific aspects of these lower extremity ulcers and their relevancy and impact on the geriatric population. The top five main results of this study can be summarized as follows. (1) Venous ulcers, caused by inflammatory processes secondary to venous reflux and hypertension, are the most common chronic leg ulcer in the geriatric population. (2) Arterial-ischemic ulcers are mainly due to lower extremity vascular disease, which itself tends to increase with increasing age setting the stage for an age-related increase in leg ulcers. (3) Persons with DM are at increased risk of developing foot ulcers mainly due to neuropathy and localized ischemia, both of which tend to increase with advancing age. (4) In geriatric patients with leg ulcers, it is important to rule out vasculitis or malignancy as causes. (5) Treatment is best made on a case-by-case basis, considering the patient's underlying condition, comorbidities, overall health status, and life expectancy.

## Introduction and background

Leg ulcers are a significant worldwide healthcare burden that occur frequently in the elderly [1-3]. A reported incidence of venous leg ulcers (VLU) in those 65 and older (geriatric) is as great as 4-5% with about 85% of all leg ulcers related to venous disease [4]. The quality of life of those living with leg ulcers is demonstrably reduced [5,6]. Early diagnosis and targeted treatment are important to minimize the overall leg ulcer burden for older adults [7-11]. The focus of this report is on the major types of leg ulcers: venous, arterial, and neuropathic. The goal of this paper is to characterize and discuss the general and specific aspects of these lower extremity ulcers and their relevancy and impact on the geriatric population.

## Review

This review is in part based on information derived from an analysis of published material obtained via literature searches of four major electronic databases and in part based on professional experiences of the senior author (HNM). The databases searched were PubMed, Web of Science, EMBASE, and Biomedical Reference Collection: Comprehensive. The search term strategy for each of these was the same as follows. The title terms used were “leg ulcer*”, “foot ulcer*” “venous ulcer*”, “arterial ulcer*”, “neuropathic ulcer*” and “diabetic ulcer*”, and “ischemic ulcer*”. The asterisk served as a wild card. These phrases were individually searched when combined (AND condition) with the following terms if they appeared anywhere in the manuscript: elderly or aged or older or geriatric. Retrieved titles were first screened for potential relevance followed by an abstract review for further clarifications if warranted by the title. Articles that were deemed relevant were retrieved and reviewed. In some cases, the bibliography of the retrieved articles provided additional sources.

Venous ulcers

**Figure 1:**
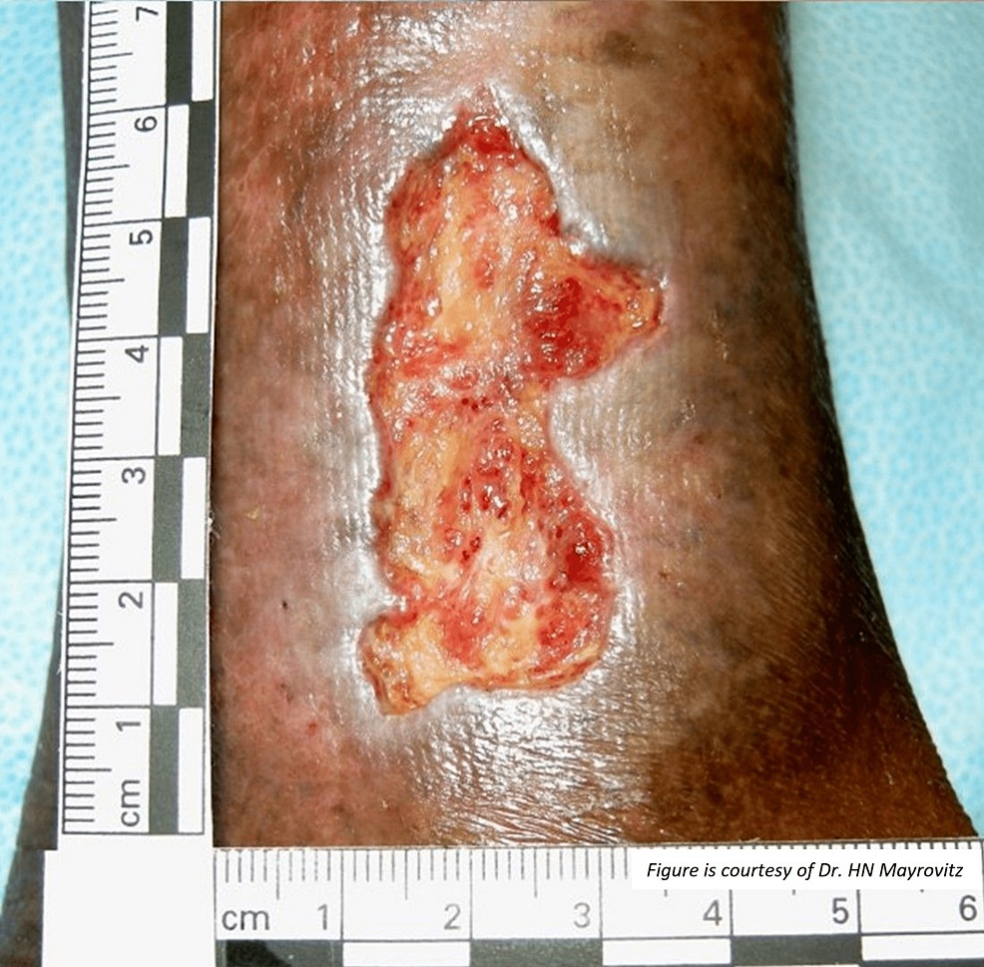
Venous ulcer located on the lateral gaiter area These ulcers typically have an irregular shape and characteristic wound bed granulation tissue and surrounding tissue hyperpigmentation. The figure is provided as a courtesy of Dr. HN Mayrovitz.

In Australia, France, Germany, Italy, Spain, the United Kingdom, and the United States combined [12], about 80% of lower extremity wounds are venous ulcers with nearly 95% of them located in the gaiter area, referring to the area between the knee and the ankle with variable areas and shapes [13]. An example of a venous ulcer located in the gaiter area is shown in Figure 1. This figure demonstrates some of its more common features, typically a shallow ulcer with irregular margins, often with surrounding hyperpigmentation. The VLU recurrence rate once healed is variable, but an analysis of nearly 400 patients indicates that a recurrence by 12 months was between 50-55% with rates being somewhat predictable based on risk classification [14].

Age-Related Incidence

Since venous disease increases with age, the challenges of VLU are increased in the geriatric population [2]. Previous leg injuries, deep vein thrombosis, phlebitis, obesity, and older age are all risk factors for the development of VLU [15,16]. About 20% of persons who develop venous ulcers do so prior to the age of 40, and the rest (a majority) develop venous ulcers after the age of 40 with about 85% of venous ulcers occurring in the geriatric population [17]. Furthermore, 40% of patients who develop VLU have a history of deep vein thrombosis and a diagnosis of chronic venous insufficiency (CVI). Twenty percent of patients with venous disease also have some arterial disease with the risk of VLU increasing with age affecting 2% of persons greater than age 80 [18], with a reported prevalence as high as 5% in the geriatric population [19].

Age-Related Risk Factors

Age-specific risk factors that contribute to this prevalence in older adults include endothelial dysfunction, frailty, and immobility [20]. In older adults, endothelial dysfunction manifests as reduced vasodilation reserve, increased prothrombotic environment, and decreased anticoagulant properties. These changes increase the risk of deep vein thrombosis in older adults and subsequently its dermatological manifestation as VLU. Similarly, immobility increases VLU risk through changes in venous hemodynamics. Since the geriatric population is most at risk of experiencing long-term immobility, there is a greater risk of developing venous ulcers. Additional age-related differences that impact the nature of VLU have been reported [21].

Causation and Healing

VLU development is affected by venous reflux and venous hypertension due to the incompetence of deep and communicating vein valves and thrombosis of deep vein segments [22]. The exact pathophysiology of VLU is not fully elucidated; however, several theories and hypothetical models have been described [23-25]. The “white cell trapping” theory has been put forward that describes a release of free radicals that cause tissue death [26]. Such microvascular entrapment of neutrophils has been reported [27]. Although the path from venous hypertension to VLU evolution of VLU is not fully understood, contributory factors likely include inflammatory processes, intercellular and vascular adhesion molecule upregulation, protein-rich edema, leukocyte trapping, oxygen deprivation, and microcirculatory deficits. Based on a re-analysis of VLU, it was reported that the main causes for the absence of VLU healing by 12 weeks of treatment were factors related to the ulcer itself, with larger ulcer size, exudate, calf circumference, and ulcer duration [28].

Venous Valve Insufficiency in Relation to VLU

**Figure 2:**
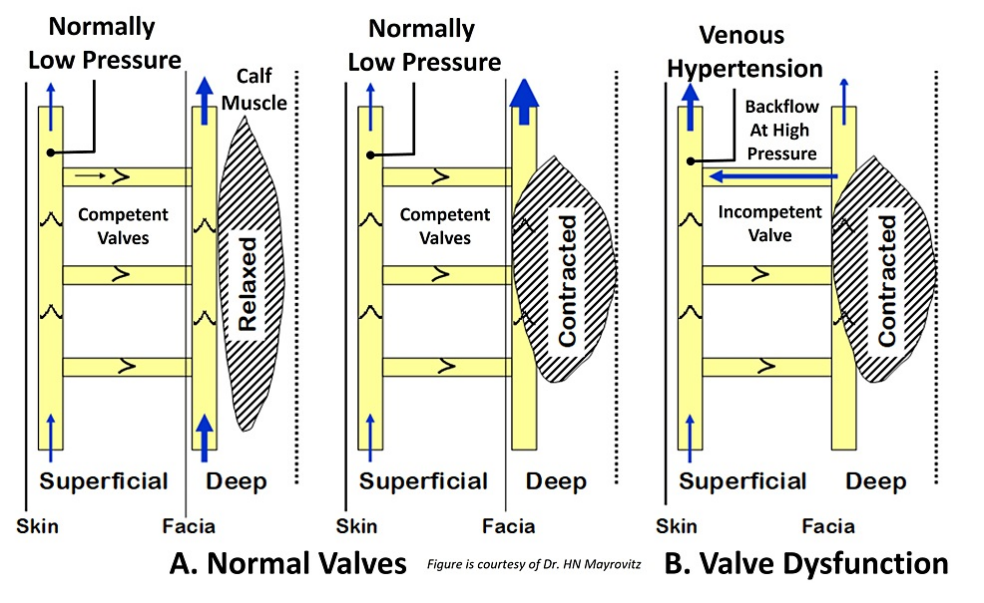
Schematic of the impact and hemodynamics of incompetent venous valves The normally low pressure experienced by the superficial veins is subject to high pressures in the presence of valve incompetency as shown in part B. This elevated pressure is not well tolerated and causes venous injury that triggers a sequence of events that may lead to the development of a venous ulcer. This figure is provided as a courtesy of Dr. HN Mayrovitz.

Factors that impact the development of CVI, a condition that is most often associated with venous hypertension that itself increases with age, include (1) dysfunction of valves in superficial and/or communicating veins, (2) dysfunction of valves located in the deep venous system, (3) obstruction to outflow from the deep venous system, and (4) dysfunction or failure of the calf muscle pump. The progression to venous ulceration is most often precipitated by the reverse flow in medial calf perforating veins that gives rise to pressure-induced venous injury, tissue damage, and skin breakdown. The blood flow path process is schematically illustrated in Figure 2, in which the normally low-pressure superficial veins become exposed to the high pressures induced by the reverse flow pathways associated with incompetent valves.

**Figure 3:**
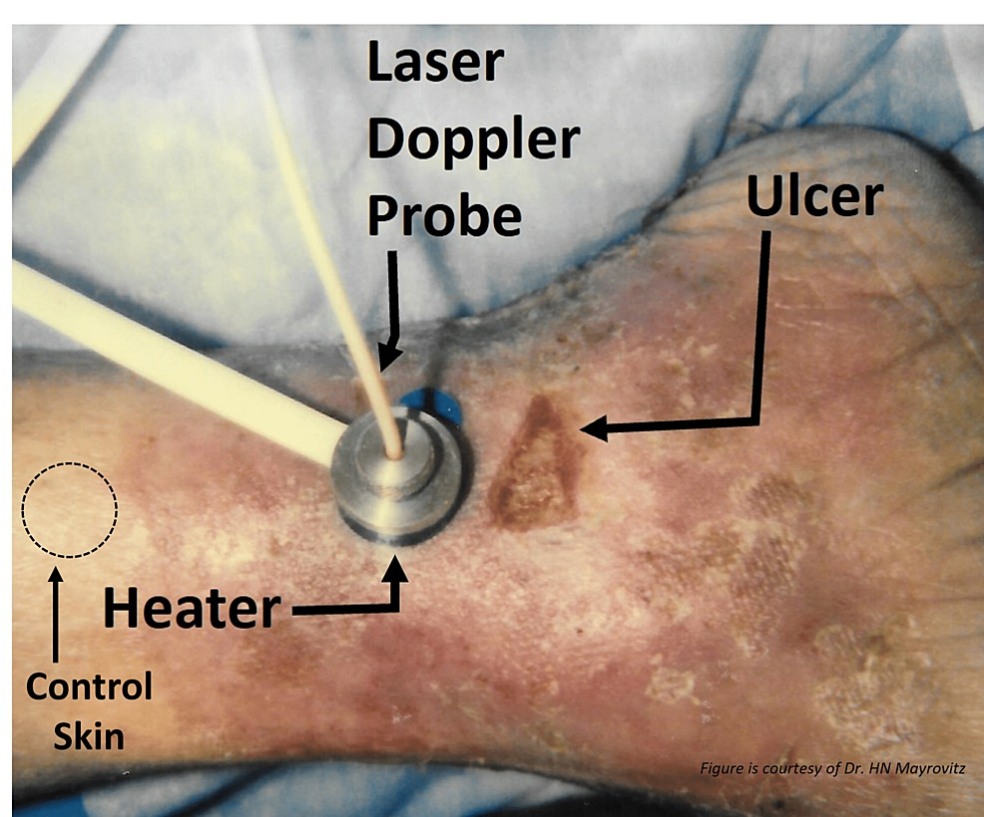
Periulcer skin blood perfusion measurement A laser Doppler probe is fitted through a concentric hole in the heater that is in contact with the skin. Localized heating produces an increase in microvascular perfusion in the healthy skin but with a different pattern in the peri-wound skin as shown in Figure 4. This figure is provided as a courtesy of Dr. HN Mayrovitz.

Concomitant with the tissue injury are inflammatory processes, increased vascular permeability, and edema and/or lymphedema. In Figure 3, an example of a venous ulcer is shown in which skin blood perfusion is being measured in the periulcer region using laser Doppler.

**Figure 4:**
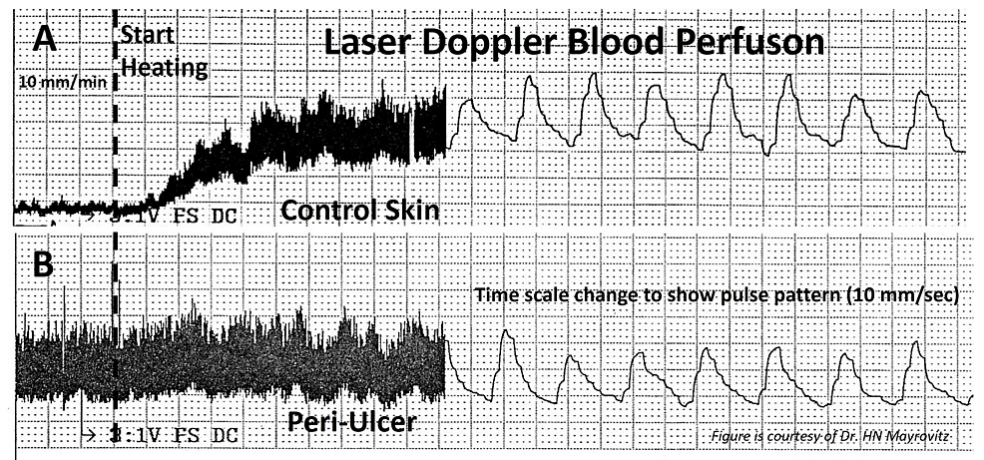
Skin blood perfusion responses to heating in healthy vs. periulcer skin The responses show a normal response to localized heating (A) and a common finding associated with venous ulcers (B). In part B, an elevated periulcer basal resting perfusion is noted with little if any microvascular reserve when stimulated with heat. In contrast, in the control skin as shown in part A, a normal active hyperemia is noted in response to the heating. This figure is provided as a courtesy of Dr. HN Mayrovitz.

The initial perfusion measurement is made at a skin temperature of 35^o^C, and then the tissue is locally heated to 44^o^C with typical responses as shown in Figure 4. The responses shown in Figure 4 demonstrate a common finding for ulcers of venous origin and an elevated periulcer basal resting flow with little if any vascular reserve when stimulated with heat as shown in part B but with normal responses in the healthy control skin as shown in part A.

Arterial ischemic ulcers

**Figure 5:**
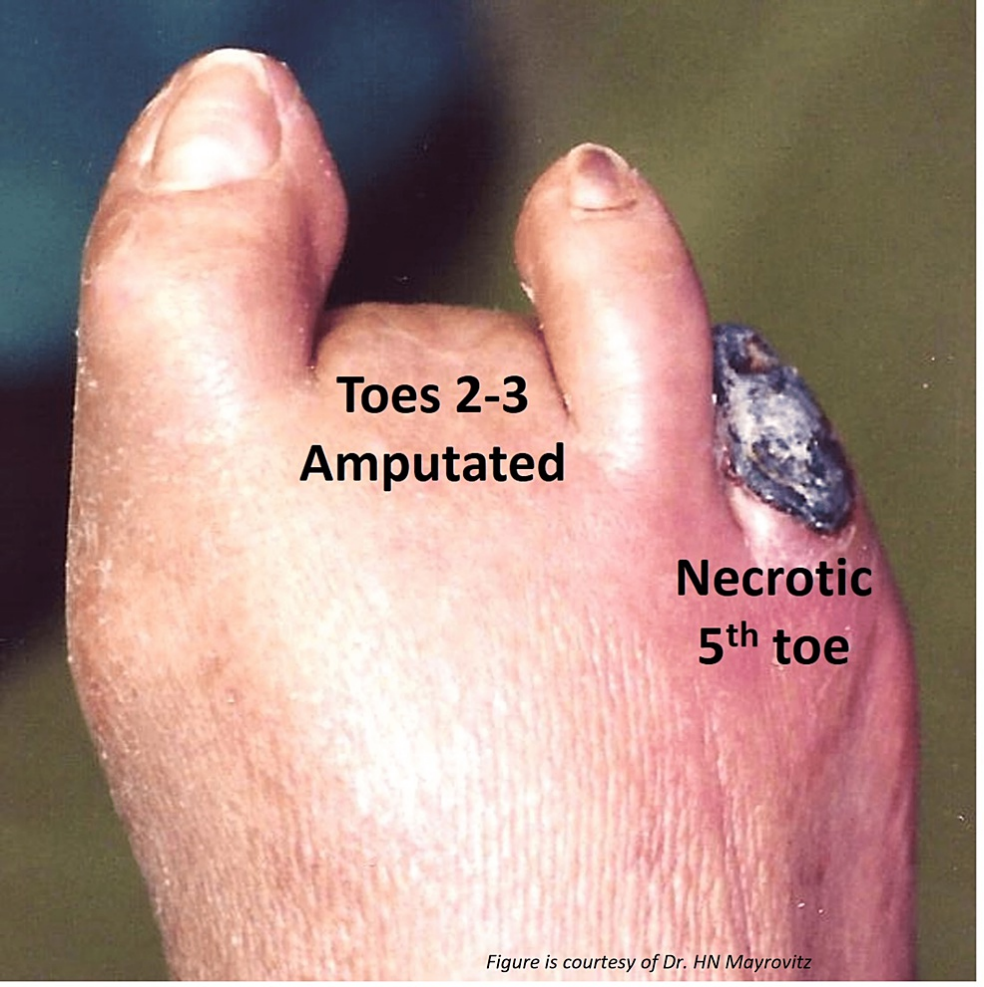
Some aspects of an arterial ulcer A patient with critical ischemia due to significant PAD in whom toes 2-3 were previously amputated and toe 5 is necrotic. This figure is provided as a courtesy of Dr. HN Mayrovitz.

Arterial Ischemic ulcers compose about 5-20% of all nonhealing lower extremity ulcers [29]. Its most common predisposing condition is an advanced peripheral vascular disease affecting lower extremity arteries that supply the leg and foot [30]. Other risk factors include atherosclerosis, hypertension, diabetes, and atrial fibrillation, all of which are more prevalent in the geriatric population [31]. Arterial ulcers are commonly located on the leg or foot area but have features quite distinct from VLU with an illustration of an arterial ischemic ulcer in Figure 5.

Age-Related Factors

Arterial ulcers are caused by inadequate tissue blood perfusion of the lower extremities [32]. These ulcers often occur in areas that normally experience external pressure or trauma often located at toes and malleolar areas, but any pressure point may be at risk in the presence of reduced blood perfusion [33]. Given the impact of aging on changes in lower extremity blood flow, such ulcers are likely to be more prevalent in the geriatric population, not because of their age, but because the prevalence of vascular disease is far more likely in this age group [34]. These ulcers are difficult to heal in the absence of a restoration of blood flow, which itself may be difficult to accomplish due to the presence of comorbid conditions, especially in the absence of surgical correction [35].

Arterial ulcers are associated with conditions in which the arterial blood flow to a tissue region is chronically less than needed for adequate tissue nourishment and metabolic needs. These ulcers are difficult to heal, especially in the absence of procedures to augment blood flow to the region. They are often painful and cause sleep disturbances, more frequent among the aged [9]. The pain associated with the ischemic ulcer has been described as a combination of nociceptive and neuropathic pain [36]. A common etiology for limitation in blood flow is the presence of lower extremity peripheral arterial disease due to numerous risk factors, most increasing with age, including atherosclerosis, diabetes, bed-bound status, and the length of the bedfast period [37]. Bedfast refers to the inability to leave the bed or being bedbound due to illness or disability. In addition to the higher likelihood of being bedfast with age, there is also an age-related higher risk of developing both atherosclerosis and diabetes, making the geriatric population at higher risk for the development of ischemic ulcers [38,39].

Neuropathic ulcers

**Figure 6:**
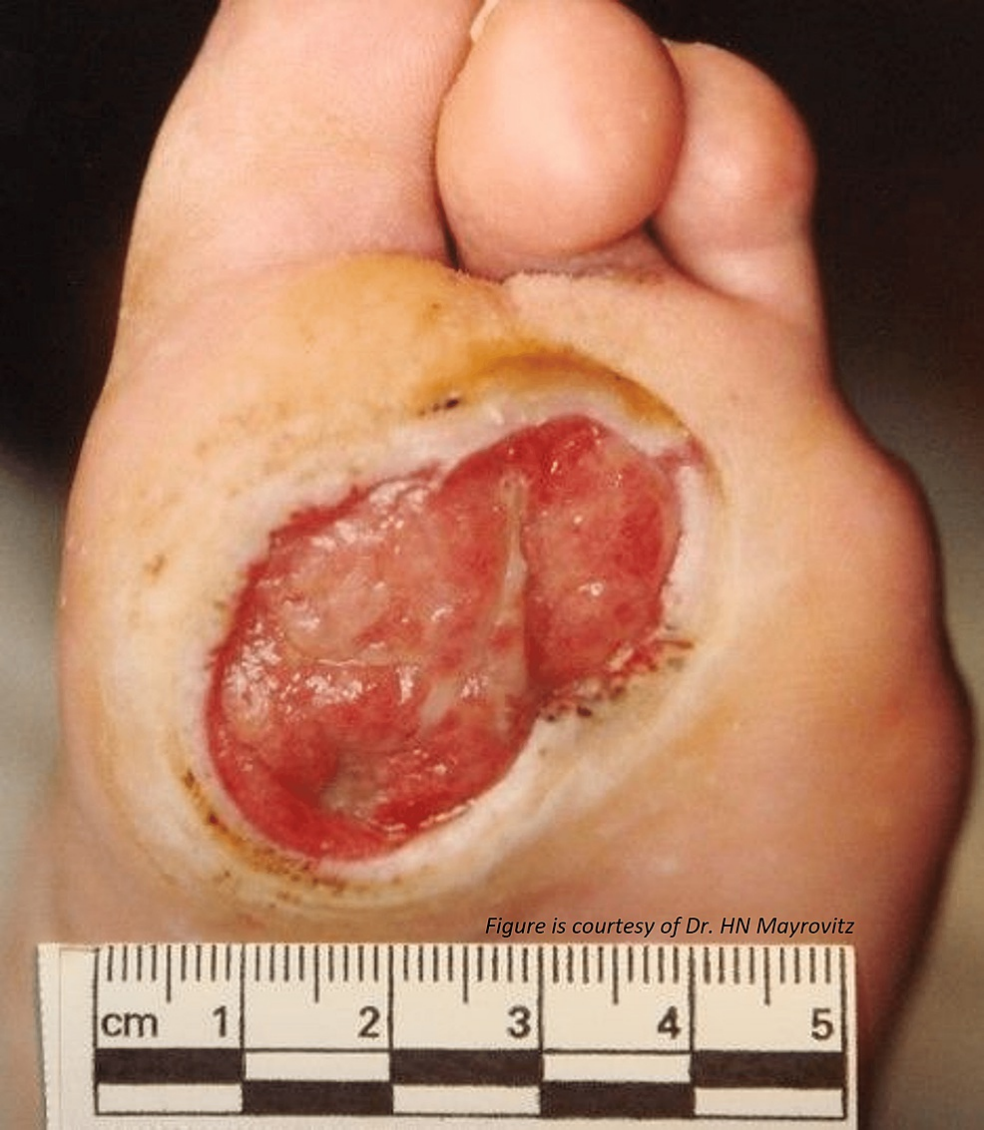
A common site of a plantar diabetic neuropathic ulcer This figure is provided as a courtesy of Dr. HN Mayrovitz.

Diabetes-related skin ulcers in persons with diabetes mellitus (DM) are generally at increased risk of lower extremity ulcers due to multiple predisposing factors, including neuropathy and localized ischemia [40]. These ulcers tend to be referred to as diabetic foot ulcers (DFU), an example of which is shown in Figure 6.

Ulcer Incidence and Complications

Statistics indicate that 15-25% of persons with DM will get a DFU [41], with an annual incidence rate of 2- 4% [42]. A recent evaluation of 3,159 patients with DM in Afghanistan found that 9.2% of them had DFU [43]. In the United States, diabetic-related nonhealing ulcers account for 140,000 extremity amputations per year [44] with an annual amputation incidence rate between 0.5% and 0.8% amputations per patient-year. In a 10-year study, the incidence of unilateral lower limb amputation for persons with DM was between 195 and 197 per 100 000 per year [41]. The disease burden of neuropathic ulcers disproportionately affects racial and ethnic minority groups [45], with Native Americans, African Americans, and Hispanics having greater rates of DM and its complications compared to Whites. These groups were also reported as less likely to undergo limb salvage than their White counterparts [46]. Other social determinants of health that are reported to impact glycemic control and ultimately health outcomes include food insecurity, neighborhood safety, perceived stress, depression, self-efficacy, and perceived discrimination [45]. People living in rural areas of the United States, as well as people who live in the South, are reported to have higher rates of lower limb amputation compared to those who live in the Northeast [47]. The pooled global prevalence of DFU has been reported to be 6.3% with the greatest prevalence in North America at 13.0% [48].

Age-Related Issues

Persons with DM who have a DFU tend to be older and have more co-present conditions including smoking history and hypertension than those without FU. One study investigated the possible relationship between DFU healing outcomes and other parameters including age and initial ulcer size in 194 patients with DFU [49]. In that study, about two-thirds of ulcers were of neuropathic origin, with most on the forefoot. Although healing time correlated with initial ulcer area, there was no detectible age-dependence in their study group who were 56.6 ± 12.6 years of age. A more recent study evaluated the impact of older age ( ≥ 75 years) on the healing rates of 1,008 patients with DM and DFU [50]. They reported that despite significant comorbidities, including 93% with neuropathy, healing was achieved in 84% of surviving patients without major amputations. In another age comparison study, the healing rates of DFU of 684 Chinese patients with DM were found to be similar between patients < 65 years and those ≥ 65 years [51]. In a study of 435 patients with diabetic FU, no essential age-related differences in ulcer presentation above and below the age of 65 were found [52]. Thus, although age may be a risk factor for developing DM-related ulcers attributable to factors such as cardiovascular and neuropathic comorbidities, age itself may not fully determine ulcer healing potential. This highlights the importance of prioritizing risk reduction in older persons to help prevent negative outcomes of DM-related ulcers [50].

Diabetes-related risk factors

Peripheral arterial disease, peripheral neuropathy, incapacity for foot self-examination, poor glycemic control, and anemia are all significant risk factors for DFU [53]. The higher likelihood of peripheral arterial disease and the presence of microvascular deficits in diabetes [54] increase the likelihood of vascular ischemia, tissue breakdown, and ulcer formation. These causative factors are more likely present in the geriatric population [55]. The risk factors for developing DFU in patients with diabetes include older age, increased BMI, duration of diabetes, and comorbidities that include neuropathy, nephropathy, or retinopathy [56]. When sensory neuropathy is present, normal pressure/pain signals are diminished or absent, removing warning signs or symptoms of tissue injury. The presence of visual impairment in some patients with diabetes makes it difficult to recognize the presence of DFU, as they are also often painless due to co-existing neuropathy.

Delayed Healing Issues

Ulcers in diabetic patients are also more difficult to heal due to reduced blood flow and wound oxygenation [57,58]. Healing time also depends on ulcer location, with ulcers on the heel taking the longest time to heal [59]. The location of DFU in geriatric patients may differ from those of younger patients with DFU based on a study of 435 patients with DFU [52]. A recent study of 674 patients with DFU carried out in China reported no difference in healing rates between patients above and below 65 years of age with both having healing rates of about 60% [51]. However, the younger patients in that study demonstrated a number of greater risk factors. In addition, a study of the role of patient frailty on DFU healing indicated it as an independent predictor of DFU healing [60]. There is also some indication that malnutrition, an often observed finding in some geriatric patients, is a factor contributing to delayed or absent healing of DFU [61]. Most ulcers develop on the sole of the foot, with plantar ulcers often associated with neuropathy with considerable variability in shape and area [62]. In these cases, the elimination of foot pressures combined with standard wound care is indicated. The risk of recurrence of previously healed DFU is impacted by co-present peripheral arterial disease and other factors [63]. Limited data suggest that supplying external oxygen to the DFU may improve healing outcomes [64].

Other potential geriatric-related ulcers

Although venous, arterial, and neuropathic ulcers comprise the bulk of lower extremity ulcers, other skin ulcerations to be considered include those related to vasculitis, infection, and malignancy. Ulcers attributed to vasculitis are most frequently found in the lower leg in part because of the relatively reduced blood perfusion and temperature in this area and the greater likelihood of this area experiencing trauma. Vascular inflammatory processes, including those caused by arteritis, contribute to leg ulcer development by causing insufficient local blood flow and tissue oxygenation. These leg ulcers are generally difficult to heal and tend to recur [65]. Diagnostic and treatment guidelines are available [66]. Because there is an age-dependent factor in the different forms of arteritis [67-69], consideration for underlying causative arteritis for some ulcers is relevant in the geriatric population. Once formed, ulcer healing may be delayed or prevented due to the presence of cellulitis or osteomyelitis, which are common causes of nonhealing ulcers [70]. It is not clear if the incidence of these infections is more prevalent in the geriatric population, but they should be considered in all nonhealing ulcers. In addition, a nonhealing ulcer may raise the concern of the presence of a cutaneous malignancy that might be mistaken for a different form of leg ulcer [71-73]. Given the age-dependent incidence of skin cancers, a malignant leg ulcer possibility is important to consider [74-76].

General considerations in geriatric patients with leg ulcers

Leg ulcers that develop in geriatric patients may eventually progress to a chronic or nonhealing state [77]. Furthermore, complications such as cellulitis, infection, malignancy, musculoskeletal changes, erythema, and pain may impact mortality [78].

Geriatric-Related Factors

Factors such as aging skin, lack of mobility, functional states as determined by activities of daily living, nutritional and social support, and multiple pathologic comorbidities common in the geriatric population all play a significant role in the development and persistence of nonhealing ulcers [79]. Lifestyle modifications such as a well-balanced diet, cessation of smoking, and consistent physical activity are recommended to help prevent ulcer recurrence [80]. Therefore, treating nonhealing leg ulcers in the geriatric population requires a holistic, comprehensive, and multidisciplinary approach [81]. The goal of treatment includes symptom control, complication prevention, and patient quality of life improvement [81,82]. The presence of multiple comorbidities in the geriatric population warrants a thorough physical examination and risk assessment before devising a treatment plan [83]. Early detection and diagnosis are important, since a delay in treatment may likely lead to disease progression [26].

Treatment Considerations

In the geriatric population, a standard treatment for VLU consists of compression, leg elevation, and exercise [83,84]. Compression therapy with zinc paste double-wrapped bandages (Unna’s Boot) or stockings with multilayer inelastic 30-40 mmHg compression bandages, re-dressed once a week, promotes healing, reduces venous reflux, and minimizes edema [26,79,83,84]. Elderly persons with VLU when treated with Unna’s Boot have shown improved measures of well-being [85]. A comparison of moderate (35-45 mmHg) vs. higher compression pressures (> 45 mmHg) suggests that higher levels achieved better healing in a study of over 100 patients with VLU and was well tolerated in geriatric patients [86]. In a retrospective evaluation of over 500 patients with VLU, the mean time to healing was reported as three months [87]. To prevent recurrences, compression therapy should be maintained [83]. Other techniques may include topical negative pressure, therapeutic ultrasound, and laser treatment [88]. Combinations of several physical modalities have been reported to increase periulcer transcutaneous oxygen tension (TcPO2) [89], and there is a reported positive correlation between TcPO2 and ulcer healing [90]. Increasing the VLU wound bed oxygen saturation via a topical hemoglobin spray also seemed to promote improved healing [91]. A program of leg exercise combined with compression also improved TcPO2 and benefited VLU healing [92]. Importantly, there is a need for focusing on and addressing patients’ underlying pathologies and comorbidities [78]. For example, in the case of VLU which remains unhealed after a year of treatment, the presence of uncorrected deep vein disease was reported as a main factor [93]. Arterial ulcers do not respond well to pharmaceutical intervention, and treatment focuses on reestablishing perfusion to the affected area [94,95]. With respect to patients with diabetes, in a retrospective evaluation of 130 patients with DFU treated with standard care, the mean time to heal was 4.4 months, with about one-third of patients healed by 12 weeks [96]. The addition of daily foot exercises to standard treatment may improve this outcome [97]. Other treatments worthy of consideration might include hyperbaric oxygen in appropriate situations and interventional radiology with minimally invasive revascularization for improved DFU outcomes.

## Conclusions

Leg ulcers are a highly prevalent and concerning problem in the geriatric population. They are often caused by underlying conditions such as venous insufficiency, peripheral artery disease, connective tissue diseases, autoimmune conditions, and diabetes, which are more prevalent in older adults. Furthermore, geriatric patients are at a higher risk of developing complications such as infection, cellulitis, and amputation, significantly impacting their quality of life and ability to function. Early identification and proper management of these underlying conditions are important elements to achieving timely leg ulcer healing and avoidance of ulcer-related complications. To achieve this, it is useful to consider treating on a case-by-case basis, considering a patient's underlying condition, comorbidities, and overall health status. This is often best achieved by a multidisciplinary approach to address unique challenges faced by geriatric patients.

## References

[1] Malay DS, Margolis DJ, Hoffstad OJ, Bellamy S (2006). The incidence and risks of failure to heal after lower extremity amputation for the treatment of diabetic neuropathic foot ulcer. J Foot Ankle Surg.

[2] Margolis DJ, Bilker W, Santanna J, Baumgarten M (2002). Venous leg ulcer: incidence and prevalence in the elderly. J Am Acad Dermatol.

[3] Andersson E, Hansson C, Swanbeck G (1993). Leg and foot ulcer prevalence and investigation of the peripheral arterial and venous circulation in a randomised elderly population. An epidemiological survey and clinical investigation. Acta Derm Venereol.

[4] Schul MW, Melin MM, Keaton TJ (2023). Venous leg ulcers and prevalence of surgically correctable reflux disease in a national registry. J Vasc Surg Venous Lymphat Disord.

[5] Cunha N, Campos S, Cabete J (2017). Chronic leg ulcers disrupt patients' lives: a study of leg ulcer-related life changes and quality of life. Br J Community Nurs.

[6] Meaume S, Dompmartin A, Lok C (2017). Quality of life in patients with leg ulcers: results from CHALLENGE, a double-blind randomised controlled trial. J Wound Care.

[7] Rayala BZ (2020). Skin ulcers: prevention and diagnosis of pressure, venous leg, and arterial ulcers. FP Essent.

[8] Lim CS, Baruah M, Bahia SS (2018). Diagnosis and management of venous leg ulcers. BMJ.

[9] Hellström A, Nilsson C, Nilsson A, Fagerström C (2016). Leg ulcers in older people: a national study addressing variation in diagnosis, pain and sleep disturbance. BMC Geriatr.

[10] Meyer V, Kerk N, Meyer S, Goerge T (2011). Differential diagnosis and therapy of leg ulcers. J Dtsch Dermatol Ges.

[11] Hayes S, Dodds SR (2003). The identification and diagnosis of malignant leg ulcers. Nurs Times.

[12] Kolluri R, Lugli M, Villalba L (2022). An estimate of the economic burden of venous leg ulcers associated with deep venous disease. Vasc Med.

[13] Mayrovitz HN, Smith J, Ingram C (1998). Comparisons of venous and diabetic plantar ulcer shape and area. Adv Wound Care.

[14] Finlayson KJ, Parker CN, Miller C (2018). Predicting the likelihood of venous leg ulcer recurrence: the diagnostic accuracy of a newly developed risk assessment tool. Int Wound J.

[15] Nelson EA, Bell-Syer SE, Cullum NA (2000). Compression for preventing recurrence of venous ulcers. Cochrane Database Syst Rev.

[16] Collins L, Seraj S (2010). Diagnosis and treatment of venous ulcers. Am Fam Physician.

[17] Nelzén O, Bergqvist D, Lindhagen A, Hallböök T (1991). Chronic leg ulcers: an underestimated problem in primary health care among elderly patients. J Epidemiol Community Health.

[18] Callam MJ, Ruckley CV, Harper DR, Dale JJ (1985). Chronic ulceration of the leg: extent of the problem and provision of care. Br Med J (Clin Res Ed).

[19] Lautenschlager S, Eichmann A (1999). Differential diagnosis of leg ulcers. Curr Probl Dermatol.

[20] Engbers MJ, van Hylckama Vlieg A, Rosendaal FR (2010). Venous thrombosis in the elderly: incidence, risk factors and risk groups. J Thromb Haemost.

[21] Klein A, Ennis W, Fukaya E (2023). Characteristics of venous leg ulcer patients at a tertiary wound care center. J Vasc Surg Venous Lymphat Disord.

[22] Gschwandtner ME, Ehringer H (2001). Microcirculation in chronic venous insufficiency. Vasc Med.

[23] Vasudevan B (2014). Venous leg ulcers: pathophysiology and classification. Indian Dermatol Online J.

[24] Comerota A, Lurie F (2015). Pathogenesis of venous ulcer. Semin Vasc Surg.

[25] Raffetto JD, Ligi D, Maniscalco R, Khalil RA, Mannello F (2020). Why venous leg ulcers have difficulty healing: overview on pathophysiology, clinical consequences, and treatment. J Clin Med.

[26] Brem H, Kirsner RS, Falanga V (2004). Protocol for the successful treatment of venous ulcers. Am J Surg.

[27] Robles-Tenorio A, Lev-Tov H, Ocampo-Candiani J (2022). Venous leg ulcer. StatPearls. Treasure Island.

[28] Weller CD, Bouguettaya A, Team V, Flegg J, Kasza J, Jayathilake C (2020). Associations between patient, treatment, or wound-level factors and venous leg ulcer healing: wound characteristics are the key factors in determining healing outcomes. Wound Repair Regen.

[29] Mekkes JR, Loots MA, Van Der Wal AC, Bos JD (2003). Causes, investigation and treatment of leg ulceration. Br J Dermatol.

[30] Spentzouris G, Labropoulos N (2009). The evaluation of lower-extremity ulcers. Semin Intervent Radiol.

[31] Weir GR, Smart H, van Marle J, Cronje FJ (2014). Arterial disease ulcers, part 1: clinical diagnosis and investigation. Adv Skin Wound Care.

[32] Grey JE, Harding KG, Enoch S (2006). Venous and arterial leg ulcers. BMJ.

[33] London NJ, Donnelly R (2000). ABC of arterial and venous disease. Ulcerated lower limb. BMJ.

[34] Dinenno FA, Jones PP, Seals DR, Tanaka H (1999). Limb blood flow and vascular conductance are reduced with age in healthy humans: relation to elevations in sympathetic nerve activity and declines in oxygen demand. Circulation.

[35] Greer N, Foman NA, MacDonald R, Dorrian J, Fitzgerald P, Rutks I, Wilt TJ (2013). Advanced wound care therapies for nonhealing diabetic, venous, and arterial ulcers: a systematic review. Ann Intern Med.

[36] Kogure T, Sumitani M, Abe H (2017). Ischemic ulcer pain is both nociceptive and neuropathic pain based on a discriminant function analysis using the McGill pain questionnaire. J Pain Palliat Care Pharmacother.

[37] Okuwa M, Sanada H, Sugama J, Inagaki M, Konya C, Kitagawa A, Tabata K (2006). A prospective cohort study of lower-extremity pressure ulcer risk among bedfast older adults. Adv Skin Wound Care.

[38] Head T, Daunert S, Goldschmidt-Clermont PJ (2017). The aging risk and atherosclerosis: a fresh look at arterial homeostasis. Front Genet.

[39] van Herpt TT, Ligthart S, Leening MJ (2020). Lifetime risk to progress from pre-diabetes to type 2 diabetes among women and men: comparison between American Diabetes Association and World Health Organization diagnostic criteria. BMJ Open Diabetes Res Care.

[40] Clayton W, Jr. Jr., Elasy TA (2009). A review of the pathophysiology, classification, and treatment of foot ulcers in diabetic patients. Clin Diabetes.

[41] Yazdanpanah L, Shahbazian H, Nazari I (2018). Incidence and risk factors of diabetic foot ulcer: a population-based diabetic foot cohort (ADFC study)-two-year follow-up study. Int J Endocrinol.

[42] Crawford F, McCowan C, Dimitrov BD (2011). The risk of foot ulceration in people with diabetes screened in community settings: findings from a cohort study. QJM.

[43] Samad Omar A, Ahmad Faiz K, Mir Islam Saeed K, Ahmad Humayoun F, Safi K (2023). Epidemiologic and clinical characteristics of diabetic foot ulcer among patients with diabetes in Afghanistan: an IDF supported initiative. Diabetes Res Clin Pract.

[44] Mizelle RM, Jr Jr (2021). Diabetes, race, and amputations. Lancet.

[45] Walker RJ, Strom Williams J, Egede LE (2016). Influence of race, ethnicity and social determinants of health on diabetes outcomes. Am J Med Sci.

[46] Durazzo TS, Frencher S, Gusberg R (2013). Influence of race on the management of lower extremity ischemia: revascularization vs amputation. JAMA Surg.

[47] Akinlotan MA, Primm K, Bolin JN, Ferdinand Cheres AL, Lee J, Callaghan T, Ferdinand AO (2021). Racial, rural, and regional disparities in diabetes-related lower-extremity amputation rates, 2009-2017. Diabetes Care.

[48] Zhang P, Lu J, Jing Y, Tang S, Zhu D, Bi Y (2017). Global epidemiology of diabetic foot ulceration: a systematic review and meta-analysis. Ann Med.

[49] Oyibo SO, Jude EB, Tarawneh I, Nguyen HC, Armstrong DG, Harkless LB, Boulton AJ (2001). The effects of ulcer size and site, patient's age, sex and type and duration of diabetes on the outcome of diabetic foot ulcers. Diabet Med.

[50] Gershater MA, Apelqvist J (2021). Elderly individuals with diabetes and foot ulcer have a probability for healing despite extensive comorbidity and dependency. Expert Rev Pharmacoecon Outcomes Res.

[51] Shi L, Xue J, Zhao W (2022). The prognosis of diabetic foot ulcer is independent of age? A comparative analysis of the characteristics of patients with diabetic foot ulcer in different age groups: a cross-sectional study from China. Int J Low Extrem Wounds.

[52] Rosinha P, Saraiva M, Ferreira L (2022). A retrospective cohort study on diabetic foot disease: ascertainment of ulcer locations by age group. Cureus.

[53] Hokkam EN (2009). Assessment of risk factors in diabetic foot ulceration and their impact on the outcome of the disease. Prim Care Diabetes.

[54] Thiruvoipati T, Kielhorn CE, Armstrong EJ (2015). Peripheral artery disease in patients with diabetes: epidemiology, mechanisms, and outcomes. World J Diabetes.

[55] Aronow H (2008). Peripheral arterial disease in the elderly: recognition and management. Am J Cardiovasc Drugs.

[56] Tang WH, Zhao YN, Cheng ZX, Xu JX, Zhang Y, Liu XM (2023). Risk factors for diabetic foot ulcers: a systematic review and meta-analysis. Vascular.

[57] Dinh T, Elder S, Veves A (2011). Delayed wound healing in diabetes: considering future treatments. Diabetes Management.

[58] Catrina SB, Zheng X (2016). Disturbed hypoxic responses as a pathogenic mechanism of diabetic foot ulcers. Diabetes Metab Res Rev.

[59] Pickwell KM, Siersma VD, Kars M, Holstein PE, Schaper NC (2013). Diabetic foot disease: impact of ulcer location on ulcer healing. Diabetes Metab Res Rev.

[60] Maltese G, Basile G, Meehan H, Fuller M, Cesari M, Fountoulakis N, Karalliedde J (2022). Frailty is associated with impaired diabetic foot ulcer healing and all-cause re-hospitalization. J Nutr Health Aging.

[61] Lauwers P, Dirinck E, Van Bouwel S (2022). Malnutrition and its relation with diabetic foot ulcer severity and outcome: a review. Acta Clin Belg.

[62] Mayrovitz HN, Smith J, Ingram C (1997). Geometric, shape and area measurement considerations for diabetic neuropathic plantar ulcers. Ostomy Wound Manage.

[63] Khalifa WA (2018). Risk factors for diabetic foot ulcer recurrence: a prospective 2-year follow-up study in Egypt. Foot (Edinb).

[64] Niederauer MQ, Michalek JE, Liu Q, Papas KK, Lavery LA, Armstrong DG (2018). Continuous diffusion of oxygen improves diabetic foot ulcer healing when compared with a placebo control: a randomised, double-blind, multicentre study. J Wound Care.

[65] Kerstein MD (1996). The non-healing leg ulcer: peripheral vascular disease, chronic venous insufficiency, and ischemic vasculitis. Ostomy Wound Manage.

[66] Fujimoto M, Asano Y, Ishii T (2016). The wound/burn guidelines - 4: guidelines for the management of skin ulcers associated with connective tissue disease/vasculitis. J Dermatol.

[67] Gloor AD, Berry GJ, Goronzy JJ, Weyand CM (2022). Age as a risk factor in vasculitis. Semin Immunopathol.

[68] Van Hemelen M, Betrains A, Vanderschueren S, Blockmans D (2020). Impact of age at diagnosis in polymyalgia rheumatica: a retrospective cohort study of 218 patients. Autoimmun Rev.

[69] Wan J, Qi S, Liao H, Ci W, Guo Y, Wang T (2020). Comparison of clinical features at the onset of takayasu's arteritis according to age and sex. Curr Vasc Pharmacol.

[70] Sibbald RG, Orsted HL, Schultz G, Keast DH (2003). Preparing the wound bed 2003: focus on infection and inflammation. Ostomy Wound Manage.

[71] Senet P, Combemale P, Debure C (2012). Malignancy and chronic leg ulcers: the value of systematic wound biopsies: a prospective, multicenter, cross-sectional study. Arch Dermatol.

[72] Smith J, Mello LF, Nogueira Neto NC (2001). Malignancy in chronic ulcers and scars of the leg (Marjolin's ulcer): a study of 21 patients. Skeletal Radiol.

[73] Waters J, Latta A, Hartley A, Jull A (2008). Malignancy and leg ulceration in a community-based leg ulcer clinic in New Zealand. J Wound Care.

[74] Niino M, Matsuda T (2021). Age-specific skin cancer incidence rate in the world. Jpn J Clin Oncol.

[75] Armstrong BK, Cust AE (2017). Sun exposure and skin cancer, and the puzzle of cutaneous melanoma: a perspective on Fears et al. mathematical models of age and ultraviolet effects on the incidence of skin cancer among whites in the United States. American Journal of Epidemiology 1977; 105: 420-427. Cancer Epidemiol.

[76] Hussain SK, Sundquist J, Hemminki K (2010). Incidence trends of squamous cell and rare skin cancers in the Swedish national cancer registry point to calendar year and age-dependent increases. J Invest Dermatol.

[77] Allman RM (1997). Pressure ulcer prevalence, incidence, risk factors, and impact. Clin Geriatr Med.

[78] Jaul E (2010). Assessment and management of pressure ulcers in the elderly: current strategies. Drugs Aging.

[79] Gould L, Abadir P, Brem H (2015). Chronic wound repair and healing in older adults: current status and future research. J Am Geriatr Soc.

[80] Brown A (2012). Life-style advice and self-care strategies for venous leg ulcer patients: what is the evidence?. J Wound Care.

[81] Jaul E (2009). Non-healing wounds: the geriatric approach. Arch Gerontol Geriatr.

[82] Grey JE, Enoch S, Harding KG (2006). Wound assessment. BMJ.

[83] Hansson C (1994). Optimal treatment of venous (stasis) ulcers in elderly patients. Drugs Aging.

[84] Nair B (2014). Compression therapy for venous leg ulcers. Indian Dermatol Online J.

[85] Faria EC, Loiola T, Salomé GM, Ferreira LM (2020). Unna boot therapy impact on wellbeing, hope and spirituality in venous leg ulcer patients: a prospective clinical trial. J Wound Care.

[86] Karanikolic V, Binic I, Jovanovic D, Golubovic M, Golubovic I, Djindjic N, Petrovic D (2018). The effect of age and compression strength on venous leg ulcer healing. Phlebology.

[87] Guest JF, Fuller GW, Vowden P (2018). Venous leg ulcer management in clinical practice in the UK: costs and outcomes. Int Wound J.

[88] Nelson EA, Adderley U (2016). Venous leg ulcers. BMJ Clin Evid.

[89] Pasek J, Szajkowski S, Pietrzak M, Cieślar G (2023). The influence of combined physical therapy procedures on oxygen partial pressure in tissues surrounding ulcer in patients with venous leg ulcers. Int J Low Extrem Wounds.

[90] Zubair M, Ahmad J (2019). Transcutaneous oxygen pressure (TcPO(2)) and ulcer outcome in diabetic patients: is there any correlation?. Diabetes Metab Syndr.

[91] Petri M, Stoffels I, Griewank K (2018). Oxygenation status in chronic leg ulcer after topical hemoglobin application may act as a surrogate marker to find the best treatment strategy and to avoid ineffective conservative long-term therapy. Mol Imaging Biol.

[92] Mutlak O, Aslam M, Standfield N (2018). The influence of exercise on ulcer healing in patients with chronic venous insufficiency. Int Angiol.

[93] Melikian R, O'Donnell TF Jr, Suarez L, Iafrati MD (2019). Risk factors associated with the venous leg ulcer that fails to heal after 1 year of treatment. J Vasc Surg Venous Lymphat Disord.

[94] Holloway GA, Jr Jr (1996). Arterial ulcers: assessment and diagnosis. Ostomy Wound Manage.

[95] Goodfield M (1997). Optimal management of chronic leg ulcers in the elderly. Drugs Aging.

[96] Guest JF, Fuller GW, Vowden P (2018). Diabetic foot ulcer management in clinical practice in the UK: costs and outcomes. Int Wound J.

[97] Eraydin Ş, Avşar G (2018). The effect of foot exercises on wound healing in type 2 diabetic patients with a foot ulcer: a randomized control study. J Wound Ostomy Continence Nurs.

